# Injectable Thermoresponsive Microparticle/Hydrogel System with Superparamagnetic Nanoparticles for Drug Release and Magnetic Hyperthermia Applications

**DOI:** 10.3390/gels9120982

**Published:** 2023-12-15

**Authors:** Henrique Carrelo, André R. Escoval, Tânia Vieira, Mercedes Jiménez-Rosado, Jorge Carvalho Silva, Alberto Romero, Paula Isabel P. Soares, João Paulo Borges

**Affiliations:** 1 CENIMAT/i3N, Department of Materials Science, NOVA School of Science and Technology (FCT NOVA), Campus de Caparica, 2829-516 Caparica, Portugal; h.carrelo@campus.fct.unl.pt (H.C.);; 2 CENIMAT/i3N, Department of Physics, NOVA School of Science and Technology (FCT NOVA), Campus de Caparica, 2829-516 Caparica, Portugal; 3 Department of Applied Chemistry and Physics, Universidad de León, 24007 Leon, Spain; 4 Department of Chemical Engineering, Facultad de Química, Universidad de Sevilla, 41012 Sevilla, Spain; alromero@us.es

**Keywords:** hydrogels, Pluronic, microparticles, rheology

## Abstract

Cancer is a disease that continues to greatly impact our society. Developing new and more personalized treatment options is crucial to decreasing the cancer burden. In this study, we combined magnetic polysaccharide microparticles with a Pluronic thermoresponsive hydrogel to develop a multifunctional, injectable drug delivery system (DDS) for magnetic hyperthermia applications. Gellan gum and alginate microparticles were loaded with superparamagnetic iron oxide nanoparticles (SPIONs) with and without coating. The magnetic microparticles’ registered temperature increases up to 4 °C upon the application of an alternating magnetic field. These magnetic microparticles were mixed with drug-loaded microparticles, and, subsequently, this mixture was embedded within a Pluronic thermoresponsive hydrogel that is capable of being in the gel state at 37 °C. The proposed DDS was capable of slowly releasing methylene blue, used as a model drug, for up to 9 days. The developed hydrogel/microparticle system had a smaller rate of drug release compared with microparticles alone. This system proved to be a potential thermoresponsive DDS suitable for magnetic hyperthermia applications, thus enabling a synergistic treatment for cancer.

## 1. Introduction

Cancer is still one of the deadliest diseases that affect humankind. Many types of therapies can be performed to treat cancer, such as immunotherapy, radiation therapy, hormone therapy, hyperthermia, and chemotherapy. Chemotherapy has many severe side effects that decrease the patient’s quality of life [1,2]. In situ drug delivery systems (DDSs) that can be placed in tumor areas (*pre*- or *post*-tumor removal surgery) might be a possible innovative treatment option [3,4]. A DDS that can be inserted in a tumorous area and progressively release necessary bioactive agents that can treat and/or even prevent cancer reoccurrence is a possibility that needs to be explored [4]. Hydrogels are good candidates for injectable in situ DDSs [5,6,7,8]. They can be composed of multiple types of polymers, including biocompatible and biodegradable ones. Hydrogels can be stimuli-responsive, where they can change their properties and behavior in response to external stimuli, including temperature, which are known as thermoresponsive hydrogels [9]. These have been used as injectable systems in the human body since they can form a stable matrix in vivo, serve as transport for bioactive agents, and also have the advantage of adjusting their shape to the injected area [10]. One viable option to control the release profile from a hydrogel’s DDS is using polymeric microparticles that can be embedded within the structure of a thermoresponsive hydrogel to regulate and prolong the release profile of the system for one or multiple drugs [10,11,12,13].

To improve cancer treatments, magnetic nanoparticles (mNPs) can also be used in DDSs, for example, as carriers of therapeutic agents or combined with polymeric microparticles using them as carriers [14,15,16]. Magnetic nanoparticles can be sensitive to alternating magnetic fields (AMFs), for example, superparamagnetic iron oxide nanoparticles (SPIONs). With the application of an AMF, mNPs are capable of increasing the temperature to ranges that lead to the apoptosis of tumorous cells (from around 41 °C to 46 °C) [17]. These can also be used as contrast agents in magnetic resonance imaging [18]. The combination of mNPs and polymeric structures has been conducted in previous studies [16,19]. Matos et al. [20] combined mNPs with nanofibrous membranes of cellulose acetate produced by an electrospinning technique. These membranes were able to increase the surrounding temperature upon the application of an AMF and exhibited no significant cytotoxicity. Regarding the encapsulation/adsorption of mNPs into polymeric microparticles, Alpdemir et al. [21] developed alginate microparticles with mNPs for sorafenib release in cancer treatments. The mNPs conferred hyperthermic capabilities to the polymeric microparticles, leading to an increase in temperature of 4 °C. Xue et al. [22] encapsulated mNPs within alginate–chitosan microspheres for doxorubicin (DOX) release in breast cancer treatments, where the application of a remote AMF affected the DOX release, enabling the regulation of release profiles.

For a more controlled drug release, thermoresponsive hydrogels can be used for transportation and as a protective barrier that can delay the drug release from drug-loaded microparticles [10]. In our previous work [23], we developed an injectable DDS composed of alginate/gellan gum microparticles within a Pluronic thermoresponsive hydrogel. The combination of mNPs and a similar microparticle/hydrogel system can be used as a possible injectable system for drug release and magnetic hyperthermia applications in possible cancer treatments. Previous work focused on the combination of Pluronic and SPIONs [24]. Other work focused on the combination of mNPs with microparticles [25,26], but the inclusion of microparticles and SPIONs into a thermoresponsive hydrogel for a DDS has, as far as we know, not been studied.

In this work, we developed polymeric microparticles with superparamagnetic iron oxide nanoparticles (SPIONs) and then included them in a multifunctional DDS composed of a microparticle/hydrogel injectable system. We aimed to confer hyperthermic capabilities to this system by adsorbing SPIONs onto the microparticles’ surface and then using them in the injectable DDS. Thus, in this study, we developed microparticles loaded with SPIONs and microparticles that were loaded with a model drug (methylene blue (MB) [27]) separately and then combined the two batches. The two batches were mixed in a 1:1 ratio. This can be useful to alter drug and mNP quantities in future studies by altering this ratio. We used bare SPIONs and SPIONs coated with 3-aminopropyl triethoxylsilane (SPIAPTS). With this study, we developed a novel DDS that combines magnetic microparticles with thermoresponsive hydrogels and has the potential for magnetic hyperthermia applications, which makes it a potential candidate for future synergistic cancer treatments.

## 2. Results and Discussion

### 2.1. Introduction of mNPs to GG:Alg Microparticles

**Figure 1:**
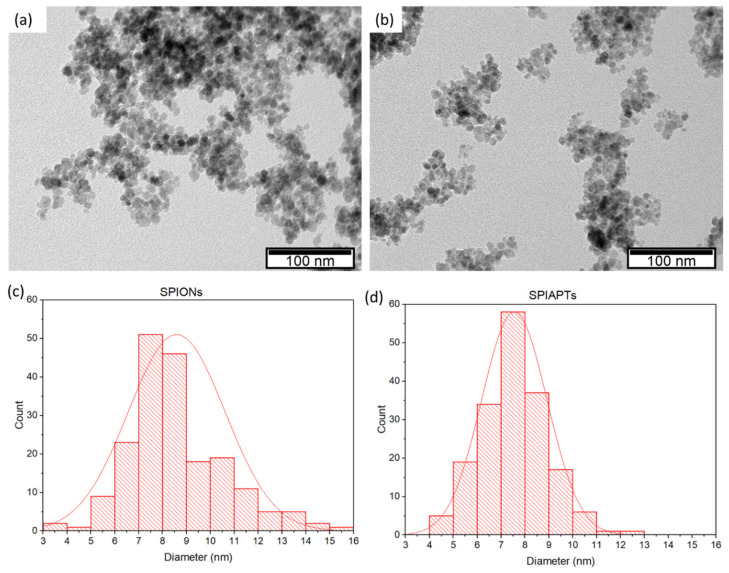
TEM analysis of (**a**) SPIONs and (**b**) SPIAPTS and their respective distribution in (**c**,**d**).

Figure 1a,b depict a TEM analysis of mNPs. Using these images, it was found that the average size of the iron core of the SPIONs was 8.59 ± 2.03 nm (Figure 1c). For SPIAPTS, the diameter was 7.55 ± 1.37 nm (Figure 1d). A difference between the average and the d(0.5) was noticed; however, they were close in both types of nanoparticles. For the two types of nanoparticles, a one-way ANOVA revealed a statistically significant difference (F(1, 370) = [32.17], *p* = 2.86 × 10^−8^). The smaller diameters obtained for the SPIAPTS might be related to the smaller aggregation that the APTES coating confers, thus allowing for a better view of the nanoparticles in TEM.

### 2.2. Magnetic GG:Alg Microparticle Characterization

**Figure 2:**
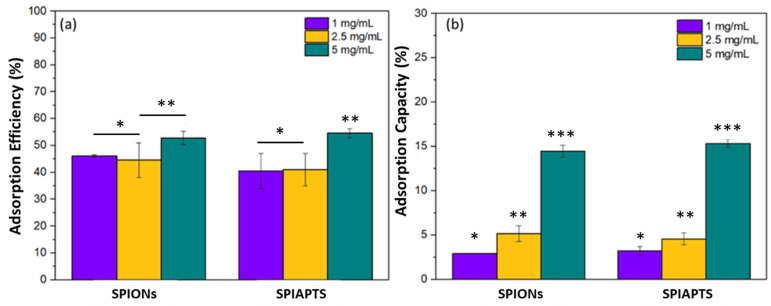
Adsorption efficiency (**a**) and adsorption capacity of SPIONs (**b**) in microparticles with and without APTES coating and at different nanoparticle concentrations (1, 2.5, and 5 mg/mL). Levels with statistically significant differences are presented by *, ** and *** above the bars (ANOVA, *p* < 0.05).

The introduction of magnetic nanoparticles to GG:Alg microparticles was performed by adsorption, where the microparticles were submerged in aqueous suspensions containing mNPs at different concentrations. The microparticles without mNPs had a diameter of 150–200 μm [23,27]. Within the mNP suspensions, the microparticles swelled, and the nanoparticles settled onto the particles’ surface. Higher adsorption efficiencies and adsorption capacities were achieved with an mNP suspension concentration of 5 mg/mL (Figure 2). The functionalization of SPIONs with APTES did not appear to affect the adsorption efficiency. Since submersion in suspensions of 5 mg/mL resulted in higher adsorption efficiencies, for the rest of the study, the default suspension to produce GG:Alg with mNPs will be 5 mg/mL.

**Figure 3:**
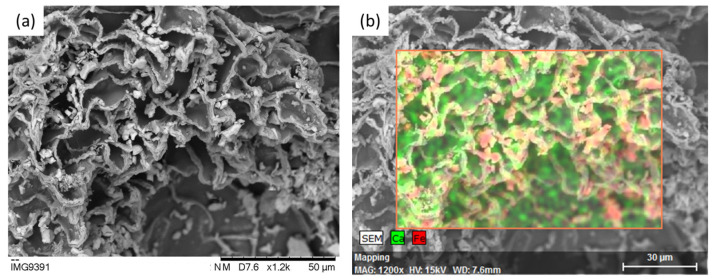

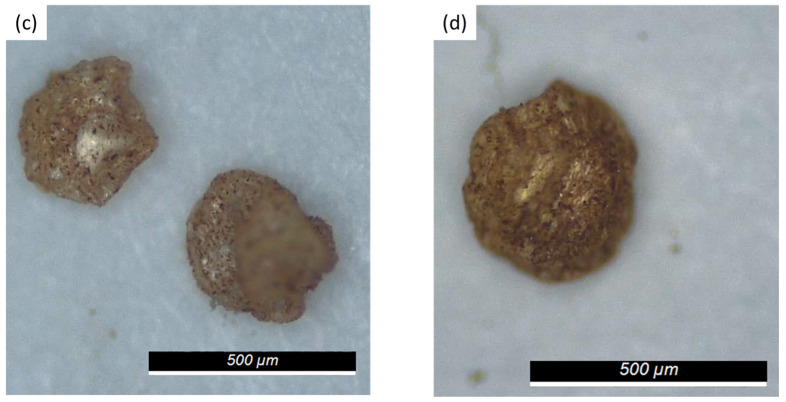
SEM analysis (**a**) and EDX analysis (**b**) of the surface of a microparticle loaded with SPIONs. In Figure (**b**), the reddish zones depict the presence of iron, and the green zones represent the presence of calcium. Microscopy analysis of (**c**) microparticles loaded with SPIONs and (**d**) microparticles loaded with SPIAPTS.

Figure 3a,b show SEM analyses of microparticles loaded with SPIONs. Their surface exhibited some agglomerations of the nanoparticles. With EDX, it is possible to see the distribution of iron throughout the surface of the microparticles. As can be observed in the image, iron is distributed throughout the surface of the microparticles, with higher contents of iron detected in certain zones on the surface, probably caused by nanoparticle clusters. The size of the GG:Alg microparticles was found to be unaltered after the adsorption of the nanoparticles (150–200 μm), as can be observed in Figure 3c,d. The APTES coating appeared to have no effect on the SPION distribution on the surface of the microparticles.

**Figure 4:**
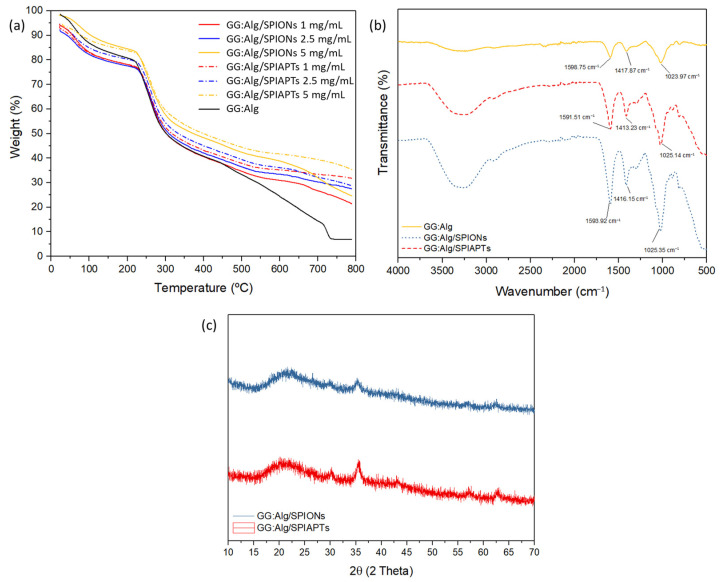
(**a**) Thermal gravimetry analysis of GG:Alg microparticles with and without mNPs with different concentrations; (**b**) Fourier-transform infrared spectroscopy of the microparticles with and without mNPs (SPIONs and SPIAPTS made with 5 mg/mL); (**c**) DRX of microparticle-loaded mNPs (SPIONs and SPIAPTS made with 5 mg/mL).

Microparticles with and without mNPs (SPIONs and SPIAPTS) were analyzed by TGA to determine their thermal stability and the effect of SPION adsorption. In all analyzed samples (Figure 4a), the first thermal event occurred at around 70 °C, characterized by a slight decrease in mass. This was associated with a loss of water from the microparticles. At around 230 °C, all samples had a significant mass loss. This was attributed to the depolymerization of gellan gum and alginate [28,29,30,31]. Subsequently, there was a steadier decline in mass percentage up to 800 °C. The microparticles containing adsorbed nanoparticles exhibited less weight loss than the microparticles without nanoparticles. This is due to the fact that the inorganic part of the nanoparticles does not degrade at the tested temperature since they are inorganic [32]. The microparticles that were submerged in a suspension of 5 mg/mL of the mNPs exhibited lower weight loss than the ones that were submerged in suspensions of 2.5 and 1 mg/mL of the mNPs. This was correlated with the loading capacity of the microparticles (Figure 2b), where higher mNP loadings were obtained at 5 mg/mL, hence the lower weight loss. It was also observed that the microparticles loaded with SPIAPTS had a slightly lower mass loss in all concentrations, possibly associated with the silane coating that did not degrade.

Regarding the FTIR results (Figure 4b), the GG:Alg microparticles without mNPs present bands around 3400 cm^−1^ and 3100 cm^−1^, attributed to the O–H groups and the –C–H stretching of gellan gum, respectively [33]. At 1598 cm^−1^, the band was attributed to –COO stretching (asymmetric), followed by –COOH stretching at 1417 cm^−1^ (symmetric) and C–O–C stretching at 1023 cm^−1^ of alginate [34]. The band of O–H stretching vibration modes at 1630 cm^−1^ might be overlapped with the –COO stretching band of gellan gum. The bands of the microparticles with SPIONs and SPIAPTS, made in suspensions of 5 mg/mL, were very similar to those of the microparticles without mNPs, except in the 700–500 cm^−1^ region in the band at 560 cm^−1^, which is attributed to the Fe–O stretching vibration mode. Microparticles submerged in suspensions of 5 mg/mL were analyzed since higher adsorption capacities were registered, thus more mNPs were adsorbed onto the GG:Alg surface. Regarding SPIAPTS, the bands around 1100 cm^−1^ and 800 cm^−1^ can be attributed to Si–O–Si and Si–O stretching vibrations, thus confirming the link between the hydroxyl group at the mNP surfaces and the alkoxysilane moieties [35,36].

XRD analysis (Figure 4c) confirms the presence of mNPs within the GG:Alg microparticles made in suspensions of 5 mg/mL. The magnetite peaks exhibit peaks at 2θ at 30.1, 35.5, 42.6, 53.6, 57.0, and 62.8, which can be assigned to diffraction of the (220), (311), (400), (422), (511), and (440) planes, respectively [37]. This is corroborated by Khalkhali et al. [38], who also registered multiple peaks for the naked SPIONs at 2θ = 18.25° (1 1 1), 30.06° (2 2 0), 35.63° (3 1 1), 43.48° (4 0 0), 53.78° (4 2 2), 57.33° (5 1 1), and 63.11° (4 4 0) peaks (with associated planes in brackets). For the two types of microparticles loaded with SPIONs and SPIAPTS, peaks at 30° (2 2 0), 35.5° (3 1 1), 57° (5 1 1), and 62.5° (4 4 0) were obtained, typical of iron oxide nanoparticles [38]. A peak with a significant width appeared at around 21°, which might have conflicted with the significant peak at 18.25° (1 1 1) of SPIONs [38]. This peak was attributed to alginate and gellan gum, and even though they have an amorphous structure, they affect the intensity and peaks in an XRD analysis, as shown by Mun et al. [39].

### 2.3. Swelling and Degradation Tests

**Figure 5:**
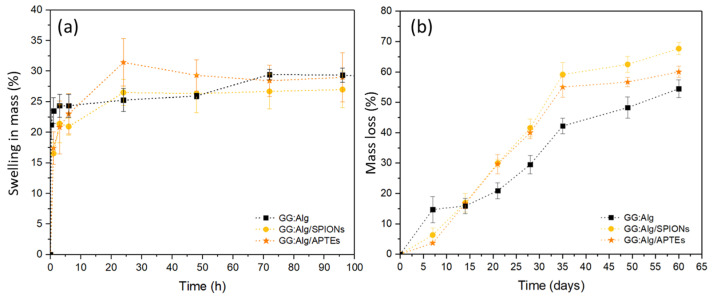
(**a**) Swelling ratio in PBS (pH 6.5) with and without mNPs (microparticles submerged in suspensions of 5 mg/mL of mNPs); (**b**) Degradation (mass loss) of microparticles with and without mNPs.

Microparticles with and without mNPs (microparticles submerged in suspensions of 5 mg/mL of mNPs) were submerged in PBS pH 6.5 (pH of tumor microenvironments), and their swelling was evaluated. All swelling indexes were similar at the end of 3 days, without significant differences caused by the mNPs (Figure 5a). Similarly, the presence of APTES at the surface of the SPIONs did not affect the microparticles’ swelling. After 3 days of swelling assays, the concentration of the mNPs in the PBS solution was measured to evaluate the number of mNPs released from the microparticles during the swelling. In PBS pH 6.5, after 3 days, GG:Alg/SPIONs had released 6.57 ±0.58 wt.% of SPIONs, and GG:Alg/SPIAPTS released 6.24 ± 1.27 wt.% of SPIAPTS into the PBS solution. Therefore, the percentage in weight of mNPs released after swelling was similar for both non-coated and coated mNPs. Regarding degradation in PBS pH 6.5, the microparticles with mNPs appeared to have greater mass loss than the microparticles without mNPs (Figure 5b). We attribute this to the conjugation between the degradation of the gellan gum/alginate structure and the release of mNPs from the microparticles; thus, a greater mass loss was observed.

### 2.4. Cytotoxicity Assays

**Figure 6:**
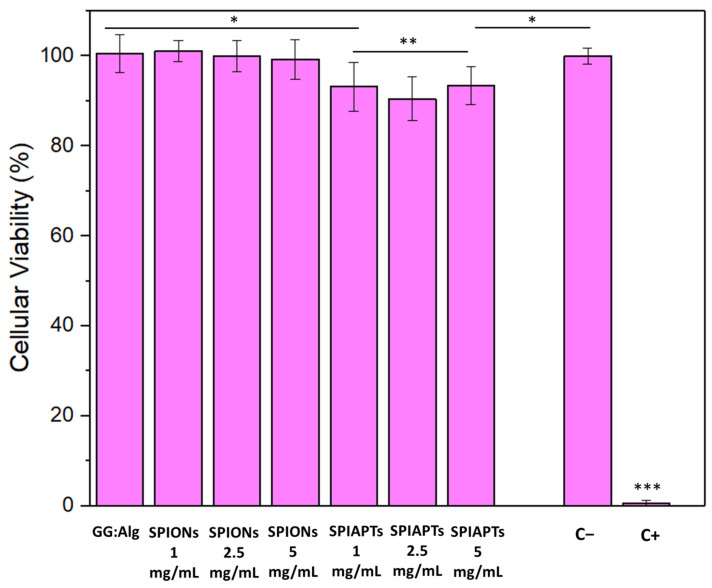
Vero cell viability (%) after 48 h of indirect exposure to the developed microparticles with and without mNPs (GG:Alg). Microparticles that were submerged in different concentrations of mNPs (1, 2.5, and 5 mg/mL) were analyzed. Data are expressed as the mean ± standard deviation of at least four experiments. C− is the negative control (no medium alterations), and C+ is the positive control (medium with 10 μL of DMSO). Levels with statistically significant differences are presented by *; ** and *** above the bars (ANOVA, *p* < 0.05).

For the cytotoxicity assays, the ISO standard 10993-5:2009 [40] was followed using Vero cells with the extract method. Figure 6 shows that, regarding cellular viability, the GG:Alg microparticles without mNPs had no cytotoxicity. Likewise, the microparticles with SPIONs did not present cytotoxicity, and the different amounts of SPIONs within the microparticles did not affect cellular viability. Similarly, the microparticles with SPIAPTS were not cytotoxic, although they presented slightly lower cellular viability compared to the others. Other studies also studied the effect that polysaccharide microparticles with iron oxide nanoparticles had on different types of cells. Alpdemir et al. [21] found that alginate microparticles with SPIONs also had no negative effects on HEPG2 liver cancer and L929 fibroblast cell lines. However, in their study, they dispersed SPIONs in a pre-crosslinked alginate solution with an initial concentration of 5 mg/mL of the SPIONs in the alginate solution. In this study, the mNPs were adsorbed to the microparticles after their crosslinking in CaCl_2_.

Before conjugating the GG:Alg microparticles with and without mNPs into a thermoresponsive hydrogel, it is fundamental to understand if they have a benign or harmful impact on the surrounding cells. Thus, cytotoxicity assays were performed, which are displayed in Figure 6. No cytotoxicity was found for the microparticles with and without mNPs. Magnetic microparticles submerged in suspensions with different concentrations of mNPs were tested and showed no significant differences between them. Although viable, the microparticles with SPIAPTS had lower cell viability than the ones with SPIONs. Cytotoxicity depends on many factors, such as the type of cell and the type of coating. For example, Alpdemir et al. [21] found that alginate microparticles containing SPIONs had no negative effects on L929 fibroblast and HEPG2 liver cancer cell lines. *t*-tests were performed, comparing each sample to a control. No significant differences were found, only with a degree of confidence of 95%, with the exception of the SPIAPTS at 2.5 mg/mL (t(2) = 0.55, *p* = 0.04). This significant difference did not alter the previously mentioned conclusions.

In our study, with the assurance that the microparticles loaded with mNPs were non-cytotoxic, we proceeded to incorporate them within the Pluronic system mixed with methylene blue (MB)-loaded microparticles. In future studies, cytotoxicity assays will be performed using cancer lines, for example, breast cancer cell lines [41].

No cytotoxicity assays were performed on the Pluronic hydrogel containing microparticles. In this study, the Pluronic hydrogel was not crosslinked, and hydrogel formation is dependent on concentration and temperature. Pluronic F127 is approved by the Food and Drug Administration (FDA) of the United States of America [42]. For example, for dental pump stem cells, Pluronic F127 was found to be non-cytotoxic [43]. Regarding breast cancer cells, in triple-negative epithelial breast cancer cell lines, a Pluronic-based hydrogel combined with hyaluronic acid was found to be non-cytotoxic [44], and only with the introduction of nanoparticles did the cytotoxicity increase. Another possibility is the mixture of Pluronic F127 and chitosan with tripolyphosphate crosslinking, as performed by García Couce et al. [45]. The addition of chitosan increased the gel time compared to the gel alone.

### 2.5. Development of a Thermoresponsive Hydrogel with Magnetic Microparticles

The optimized GG:Alg microparticles with SPIONs and APTES were mixed with plain GG:Alg microparticles at a ratio of 1:1 (wt.%). This system was analyzed via rheological and magnetic hyperthermia tests. For the release tests, plain microparticles were loaded with MB, as was previously stated. In a previous study [23], different mixtures of Pluronic F127 and Pluronic F68 were analyzed with different concentrations of GG:Alg microparticles. The system must be able to be injectable at 20 °C (operating room temperature) and be in the gel phase at 37 °C. It was found that a Pluronic mixture with 17 wt.% of Pluronic F27 and 3 wt.% of Pluronic F68 with 2 and 5 wt.% concentrations of GG:Alg microparticles meets the desired criteria. In this present study, we used a Pluronic aqueous solution with 17 wt.%:3 wt.% of F127:F68 and 2 and 5 wt.% of GG:Alg microparticles, where half were loaded with MB and the other with mNPs.

#### 2.5.1. Rheological Tests

**Figure 7:**
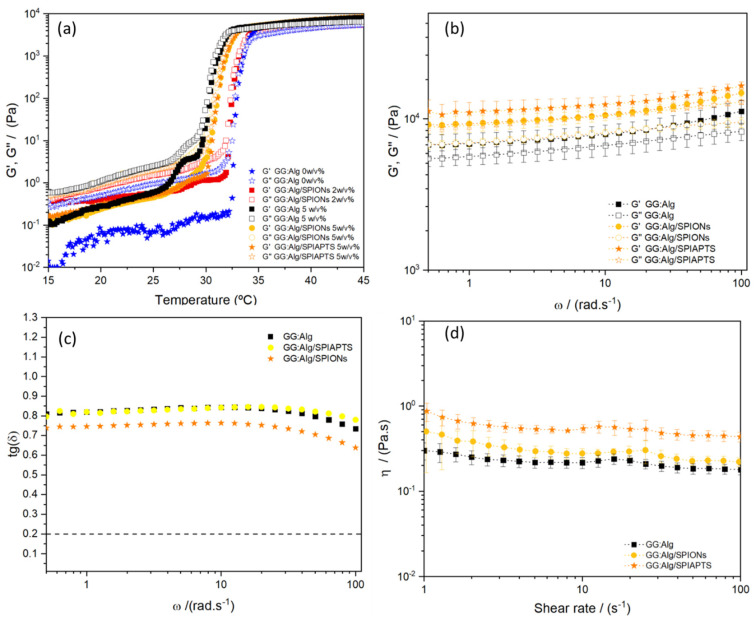
(**a**) Temperature ramp in oscillation for Pluronic systems (F127:F68—17:3) with and without microparticles. The samples with just GG:Alg refer to samples without mNPs, and the samples with GG:Alg/SPIONs and GG:Alg/SPIAPTS refer to systems with mNPs (a ratio of 1:1 of GG:Alg and GG:Alg/mNPs); (**b**) frequency sweep tests at 37 °C for similar systems (with 5 *w*/*v*% of microparticles with and without mNPs); (**c**) tg(δ) of the analyzed systems in (**b**), where a line on 0.2 was put to mark the threshold between soft gel and non-soft gel; (**d**) flow curves at 21 °C of a similar system with 5 *w*/*v*% of microparticles within the Pluronic with and without mNPs).

All systems had viscous behavior at a lower temperature since the Pluronic was in the sol state, with G″ > G′ (Figure 7a). With a temperature increase, a sol-gel transition occurred due to micellization of the PEO-PPO-PEO structure, typical of Pluronic systems [46]. The Pluronic system without microparticles had a sol-gel transition at 32–34 °C. With the addition of the microparticles at 5 *w*/*v*%, the transition temperature of the microparticle/Pluronic system decreased to around 29–32 °C. The transition temperature was not affected by the presence of mNPs (coated or not). At a 2 *w*/*v*% concentration of microparticles with mNPs, there was no significant sol-gel temperature decrease. All systems were in the sol state at 21 °C and in the gel state at 37 °C. The decrease in sol-gel temperature might be attributed to the absorption of water from the microparticles, increasing the Pluronic concentration and leading to a decrease in the sol-gel temperature. These results are in agreement with our previous study with similar microparticles and similar Pluronic hydrogels [23]. With similar microparticle concentrations (2 and 5 *w*/*v*%) but without the adsorbed mNPs, similar transition temperatures were obtained. In this study, we observed that the mNPs did not alter the sol-gel transition. Different microparticle concentrations can be used to maintain the injectability of the system by altering the Pluronic F127:F68 ratio [23].

Regarding frequency sweeps at 37° (Figure 7b), all analyzed systems were in the gel state, with G′ > G″ with a positive slope with frequency. The presence of SPIONs and SPIAPTS affected the G′ and G″. Analyzing the tg(δ) (G″/G′) (Figure 7c), it was observed that, along the studied angular frequencies, at 37 °C, the Pluronic system with microparticles (with and without mNPs) had values of tg(δ) between 0.7 and 0.8. These values reveal that the gel had high values of G″, although G′ was predominant. This indicates that the Pluronic system with microparticles at 37 °C was a weak gel with a weak elastic structure [47].

An increase in the moduli was detected with the introduction of mNPs, with the microparticles with SPIAPTS having the higher moduli. Microparticles within the Pluronic solution might act as reinforcements, leading to an increase in the resistance to the flow of the system [48]. Other interactions between the mNPs and Pluronic can also be at play. Previous studies used Pluronic F127 to functionalize iron oxide mNPs, but with previous coating agents such as oleic acid, where the hydrophobic PPO block interacted with the oleic coating of the SPIONs [24]. For example, Jain et al. [49] in FTIR studies in systems with SPIONs with and without oleic acid coating and with Pluronic showed that without oleic acid, no interaction occurred with Pluronic. While, with the oleic layer, bonding was observed in the FTIR spectra. The increased viscosity that was observed with SPIAPTS might be an interaction between the hydrophilic portions of Pluronic and the amine groups of the APTES coating; however, further studies need to be performed to understand if there are interactions between the SPIAPTS coating and the Pluronic micelles.

Regarding flow curves, at 21 °C, at a 5 *w*/*v*% concentration of the microparticles, they exhibited viscosities between 0.1 and 1 Pa·s (Figure 7d), with a slight shear-thinning behavior. However, an increase in viscosity occurred with the introduction of nanoparticles, which was more evident with the system with SPIAPTS. The same argument used for the increase in moduli is used here [48]. In the future, injectability studies will be performed [50]. In previous studies, Pluronic F127 and F68 were shown to be capable of being injected with force values within the standard ranges [50]. It is expected that the addition of microparticles alters the force, but within acceptable limits.

**Figure 8:**
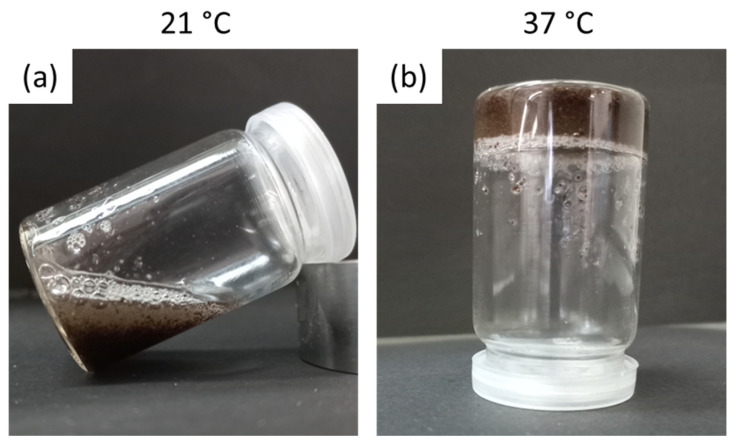
Pluronic hydrogel with GG:Alg microparticles (2 wt.%) loaded with SPIONs (GG:Alg/SPIONs) at (**a**) 21 °C and (**b**) 37 °C.

Figure 8 shows images of the proposed system with 2 *w*/*v*% of microparticles. The system is in the sol state at 21 °C (Figure 8a) and in the gel state at 37 °C (Figure 8b) according to the obtained moduli in Figure 7a.

#### 2.5.2. Magnetic Hyperthermia Tests

**Figure 9:**
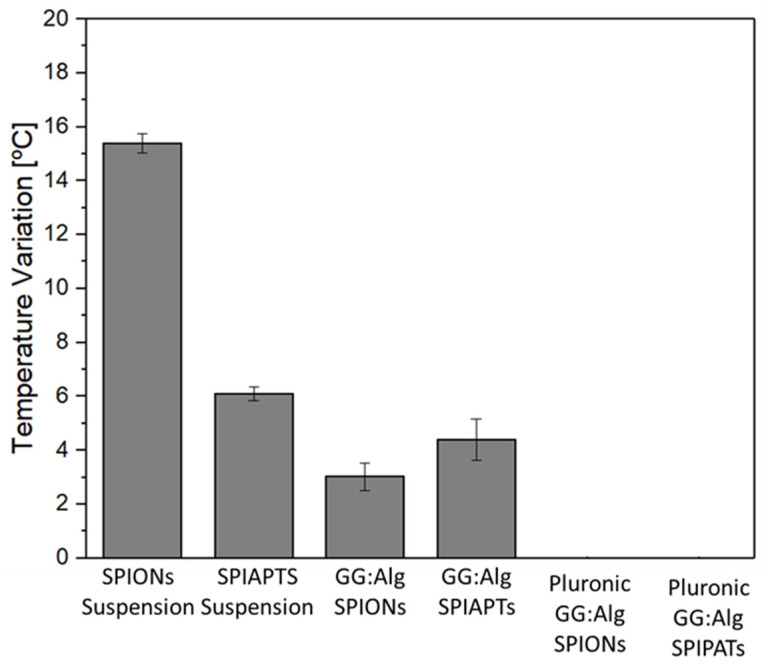
Magnetic hyperthermia study: temperature variation with the application of an alternating magnetic field (300 Gauss and 388.5 kHz) (microparticle/hydrogel system with 5 *w*/*v*%).

Figure 9 depicts the temperature variation of the systems with mNPs upon the application of an AMF. The SPION aqueous suspension had a higher temperature variation than the SPIAPTS aqueous suspension. This has been observed in previous studies [36], and it is attributed to the presence of the coating agent, where the presence of the non-magnetic APTES moieties on the mNPs’ surface can suppress the mNP relaxation mechanisms that produce heat [51]. The nanoparticles within the suspension had a higher temperature variation than the nanoparticles within the microparticles. This was attributed to the restriction of Brownian movements since the nanoparticles were immobilized at the surface of the microparticles, and the temperature increase only occurred due to Néel relaxation with the application of the AMF [52]. However, the GG:Alg microparticles with mNPs had an increase in temperature from 3 to 4 °C. Magnetic hyperthermia therapy needs to increase the tumor area temperature up to a range of 42 °C to 46 °C to cause apoptosis of the tumor cells [53]. These temperature variations are suitable for possible hyperthermic treatments. However, it is possible to alter the temperature variation of the systems by altering the concentration of mNPs in the system [20].

With the introduction of the microparticles with mNPs to the Pluronic solution and gelation at 37 °C, no temperature increase was detected. This reduced hyperthermic response has been registered in similar magnetic gels that restrain both Brownian and Néel movements and, consequently, reduce the temperature increase [54,55]. Although the mNPs generated no heat within the hydrogel in the gel state, they did produce heat without the hydrogel. Within the body, the Pluronic hydrogel will dissolve first, leaving the nanoparticles free of the gelated structure of the hydrogel. In our previous study, similar Pluronic hydrogels within PBS solutions disappeared in hours [23], while in this study, in PBS solutions, the microparticles with the mNPs lasted for over 60 days (Figure 5b). Heo et al. [55] studied dexamethasone-loaded microparticles within a Pluronic hydrogel. In in vivo trials, the Pluronic hydrogel disappeared within 6 days, leaving the microparticles exposed to the in vivo environment for longer periods. Thus, the proposed system allows for earlier drug release, followed by the possibility of magnetic hyperthermia. Table 1 presents the SAR values. The values follow the trend from the temperature variation, where a higher SAR was obtained from the free-nanoparticle suspension, with the SPION suspension having a higher energy absorption rate.

#### 2.5.3. Methylene Blue Release Assays

**Figure 10:**
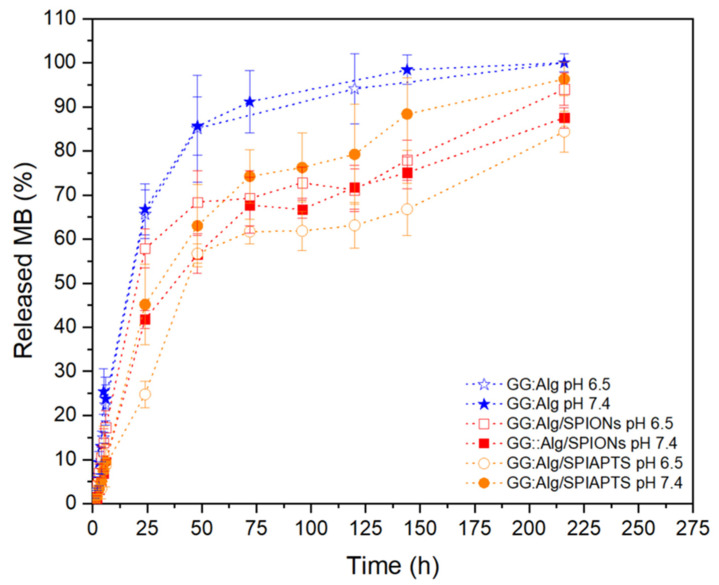
Release profiles of GG:Alg microparticles loaded with MB within a Pluronic hydrogel and with GG:Alg microparticles loaded with mNPs (the ratio of microparticles with MB and microparticles with mNPs is 1:1).

With regard to the MB release profiles, the systems with MB-loaded microparticles within the Pluronic F127:F68 (17:3) aqueous solutions with and without magnetic microparticles were analyzed (Figure 10). All systems had an earlier fast release, followed by a stabilization of the release rate. The systems without magnetic microparticles had higher quantities of released MB than the systems with magnetic microparticles. Since the microparticles with mNPs were not loaded with MB, they may have absorbed part of the released MB from the other microparticles, thereby delaying the release of MB. The systems with both types of microparticles had an earlier, faster release, followed by a stabilization phase with lower quantities of released MB than the systems with just one type of microparticle. Afterwards, the MB release continued to increase until it reached values similar to the ones obtained with just one type of microparticle. This might be attributed to a stabilization of the absorbed and released quantities of MB between the two types of microparticles, leading to more similar values to the system with just MB-loaded microparticles.

Comparing the systems with mNPs, there were no significant differences between the released MB quantities from the systems at pH 6.5 and pH 7.4. However, with SPIAPTS, the system at pH 6.5 had a lower MB release amount than the one at pH 7.4. Moreover, the release profile with SPIAPTS at pH 7.4 had a higher released quantity of MB than the ones with SPIONs, and the release profile at pH 6.5 had lower quantities of MB released. In previous work with a similar system [23], the pH did not significantly affect the release profile, similar to the systems with just SPIONs.

Mathematical modeling of the experimental data was carried out using the KP and PS models with T_lag_ to adjust to the swelling systems since these microparticles swelled (Table 2). Both models had good fittings with the obtained results, with all adjusted R^2^ > 0.95. In both models and the different release systems, all systems followed a Fickian release profile, with *n* ≤ 0.43 with the KP-T_lag_ model and *k*_1_ >> |*k*_2_| with the PS-T_lag_ model. In our previous work [23], with similar microparticles without mNPs and a hydrogel, the same release profile was obtained. This reveals that the addition of mNPs to the microparticle/hydrogel system does not alter the type of release profile of MB from the GG:Alg microparticles.

The effect of the mNPs on the release profile was assessed; however, the release profile of the microparticle/hydrogel system with the application of an AMF was not tested. However, in previous works, the application of an AMF led to a faster release of loaded drugs from microparticles with mNPs. Finotelli et al. [19] developed alginate/chitosan beads containing magnetite nanoparticles for insulin release. The application of the magnetic field increased the release profile three-fold. In another study, Xue et al. [22] produced DOX-loaded magnetic alginate–chitosan microspheres with SPIONs. The application of an alternating magnetic field also promoted a more pronounced release of DOX. With this, we suppose that a similar effect might occur in the hydrogel’s presence or when it dissolves. However, this hypothesis needs to be studied in future works.

## 3. Conclusions

The objective of this study was to develop an injectable thermoresponsive DDS with potential applications in future cancer treatments through a synergistic combination with magnetic hyperthermia. We successfully loaded mNPs onto polymeric microparticles and observed that the APTES coating on SPIONs did not affect the adsorption of mNPs onto the surface of the microparticles. The magnetic microparticles were capable of increasing the surrounding temperature upon exposure to an external AMF, with SPIONs increasing the temperature by 3 °C and SPIAPTS by 4 °C. The microparticles alone were found to be non-cytotoxic to Vero cell lines, and the addition of mNPs did not alter this finding. Nevertheless, the incorporation of magnetic microparticles into a Pluronic hydrogel did not show any temperature increase upon AMF application. Therefore, the designed system can be used for sequential treatments in two phases: burst release in the first hours after injection caused by hydrogel disaggregation, followed by slower drug release from drug-loaded microparticles combined with magnetic hyperthermia supported by magnetic microparticles. Since drug-loaded and magnetic microparticles are independent, the amount of drug and mNPs can be tailored to achieve the best synergistic cancer treatment.

## 4. Materials and Methods

All the chemical reagents used in this research work were of analytical grade and used without further purification. Pluronic F127, Pluronic F68, and high-acyl gellan gum (Phytagel) were purchased from Sigma Aldrich (St. Louis, MO, USA). Alginic acid sodium salt was purchased from BioChemica (Panreac Química SLU, Barcelona, Spain). Methylene blue (MB), in powder form, was purchased from Alfa Aesar (Haverhill, MA, USA), and calcium chloride was purchased from Carl Roth (Karlsruhe, Germany). For the mNPs, iron (III) chloride hexahydrate was purchased from Fluka (Seelze near Hannove, Germany), and iron (II) chloride tetrahydrate was purchased from ThermoFisher (Waltham, Germany). For the 3-aminopropyl triethoxylsilane (APTES) coating, ammonia (25%) was purchased from VWR International (Radnor, PA, USA), and 3-(Triethoxysiliyl)-propylamine was purchased from Sigma Aldrich.

### 4.1. Nanoparticle and Microparticle Production

The microparticles were produced from gellan gum and alginate (GG:Alg). These were produced using a coaxial airflow technique, where a mixture of polysaccharides was extruded into a CaCl_2_ bath with the aid of a parallel airflow. An aqueous solution of 2 *w*/*w*% GG:Alg (1:1 ratio) was used with an airflow of 5 L/min, a pump flow of 5 mL/h, and a nozzle-bath height of 15 cm. These parameters were established following previous work from our group [23,27]. Then, the GG:Alg microparticles were freeze-dried (Freeze Dryer—Zirbus VaCo 2 (Harz, Germany)). The dried microparticles presented a diameter between 150 and 200 μm [23].

Superparamagnetic iron oxide nanoparticles (SPIONs) were produced using a previously described co-precipitation technique [56,57]. A part of these SPIONs was conjugated with APTES, which is an aminosilane coupling agent that is used to coat the surface of nanoparticles, following a previously described method [35]. Batches with non-coated nanoparticles are referred to as SPIONs, and batches with coated nanoparticles with APTES are referred to as SPIAPTS. Both types of nanoparticles are also addressed as mNPs.

Another aim of this study was to load the GG:Alg microparticles with the nanoparticles to produce magnetic GG:Alg microparticles. SPIONs were loaded into the microparticles by swelling the GG:Alg microparticles within SPION/SPIAPTS suspensions via adsorption [20]. Freeze-dried GG:Alg microparticles were submerged in mNP suspensions with different concentrations (1, 2.5, and 5 mg/mL) for 4 days (allowing the maximum swelling of the microparticles) at 37 °C in an orbital shaker. Afterwards, the microparticles were washed for 5 min 3 times with ultrapure water to remove excess unbounded mNPs and freeze-dried.

For the drug release assays, GG:Alg microparticles without SPIONs were loaded with methylene blue (MB), according to previous work [10]. In a PBS solution with pH 7.4 and 300 μg/mL of MB, the microparticles were submerged for 4 days to absorb the MB, with an encapsulation efficiency of 77.83 (±0.56)% [10]. Then, they were washed with ultrapure water, filtrated, and freeze-dried.

### 4.2. Nanoparticle/Microparticle/Hydrogel Composite

**Figure 11:**
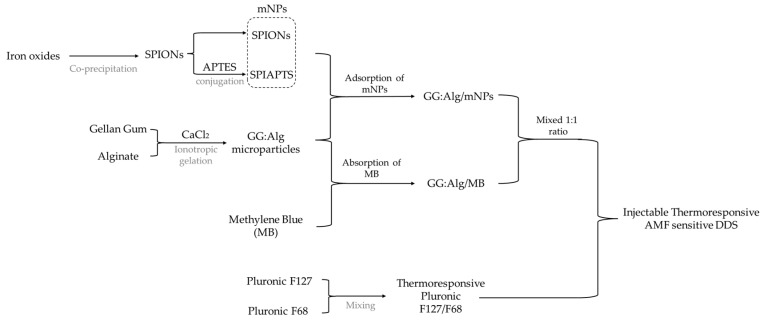
Scheme of the production of the DDS. The iron oxides used were Fe^2+^ and F^3+^.

Pluronic solutions were produced according to our previous study using a Pluronic aqueous solution (20 *w*/*w*%) with a mixture of Pluronic F127 (17 *w*/*w*%) and F68 (3 *w*/*w*%) [23]. The mixtures of Pluronic and ultrapure water were produced and left at 4 °C overnight to ensure complete dissolution. The Pluronic ratio of 17:3 provided that this solution was in the sol state at operating room temperature (21 °C) and in the gel phase at body temperature (37 °C) [23]. We aimed to produce a thermoresponsive magnetic DDS. Thus, dried microparticles loaded with MB and dried microparticles with SPIONs were mixed in a 1:1 mass ratio. This ratio was maintained throughout the study. Then, Pluronic and microparticles (with a ratio of 1:1) were mixed in the sol stage of the Pluronic. According to our previous study, a mixture of 2 *w*/*v*% to 15 *w*/*v*% of microparticles in the Pluronic hydrogel (20 *w*/*w*% aqueous solution) (with a similar F127:F68 ratio) allows the microparticle/Pluronic system to meet the above temperature criteria [23]. Figure 11 depicts the process of DDS production.

### 4.3. mNP Characterization

Transmission electron microscopy (TEM) images were obtained using a Hitachi H-8100 thermionic emission LaB (Japan).

### 4.4. Microparticle Characterization

The freeze-dried particles were analyzed using an S9 stereo optical microscope (Leica^®^, Wetzlar, Germany) and the SEM tabletop microscope TM3030 Plus (Hitachi^®^, Chiyoda, Japan). Optical microscopy images were also obtained in the transmission mode using an Olympus BX51 microscope (Olympus^®^, Tokyo, Japan), coupled with an Olympus DP73 CCD camera, and acquired with the Stream Basic v.1.9 Olympus software. A cold illumination source generated by a halogen lamp (KL 2500 LCD, SCHOTT, Mainz, Germany.) was used. All images were obtained and automatically scaled by the respective software.

TGA analyses were performed on the GG:Alg microparticles with and without mNPs to analyze any possible differences with the addition of these. All analyses were carried out with TGA-DSC—STA 449 F3 Jupiter equipment from Netzsch (Selb, Germany). They were performed in a temperature range of 25–800 °C with a 10 °C/min heating rate under a nitrogen atmosphere. FTIR analyses of the microparticles with and without nanoparticles were performed in a Perkin-Elmer Spectrum Two (Waltham, MA, USA), equipped with an attenuated total reflection cell (ATR), in the range of 4000–500 cm^−1^. DRX measurements were performed through a D81 90 powder diffractometer (Malvern Pananalytical, Worcestershire, England). X-rays at 40 kV and 30 mA impacted with a pitched angle of 0.015° and a passing time of 0.1 s on the samples, which rotated at 30 rpm.

### 4.5. Adsorption Efficiency and Adsorption Capacity

The adsorption efficiency (AE%) and adsorption capacity (AC%) were determined by adding 0.02 g of GG:Alg microparticles to 5 mL of mNP suspensions with different concentrations (1, 2.5, and 5 mg/mL) for 4 days at 37 °C in an orbital shaker to ensure maximum swelling of the microparticles. The concentration of free SPIONs in suspension was determined using the 1,10-phenantroline method [14,58] and measuring the maximum absorbance at 510 nm on a T90+ UV/VIS spectrometer (PG Instruments Ltd., Lutterworth, UK) [54,57]. AE% and AC% were determined using the following equations:

(1) $$the\;AE\left(\%\right)=\left(\frac{{ms}_{0}-{ms}_{f}}{{ms}_{0}}\right)\cdot 100;$$

(2) $$AC\left(\%\right)=\frac{m_{encap.mNPs}}{m_{GG:Alg}}\cdot 100$$

where ${ms}_{0}$ is the mass of the nanoparticles in the initial suspension, ${ms}_{f}$ is the remaining mass of the nanoparticles in the suspension after swelling of the microparticles, $m_{encap.\;mNPs}$ is the mass of the adsorbed nanoparticles in the microparticles, and $m_{GG:Alg}$ is the weight of the microparticles. Four replicas were used in each assay.

### 4.6. Swelling (S_w_) and Degradation

For the swelling studies, microparticles with mNPs, previously prepared in a 5 mg/mL suspension, were submerged in PBS solution (pH 6.5). At different times, the microparticles were removed from the PBS solution and weighed. At equilibrium, the concentration of the mNPs in the PBS solution was analyzed using the previously mentioned 10-phenanthroline method [14,58] to evaluate the nanoparticles’ concentration released during swelling. Swelling in weight (Sw) was calculated as the following equation describes:

(3) $$S_{w}=\frac{m_{tw}-m_{0}}{m_{0}}$$

where m_tw_ is the mass of the microparticles at different times after swelling, and m0 is the initial mass of the dried microparticles. For the analysis of degradation, different batches of microparticles with and without mNPs were submerged in PBS solution (pH 6.5), and, at different times, they were removed, filtered, rinsed with water, dried, and weighed.

### 4.7. Rheological Analysis

The sol-gel transition of the microparticle/Pluronic system was rheologically characterized using an AR2000 stress-controlled oscillatory rheometer (TA Instruments, New Castle, DE, USA). The analyses were performed using a plate–plate smooth geometry (dia: 40 mm) with a selected gap of 2 mm. This gap was chosen to accommodate the size of the microparticles. Firstly, strain sweep tests were performed to determine the linear viscoelastic range. From these, a strain of 2% was selected for the rest of the tests. Temperature ramps were performed between 15 and 45 °C at a constant frequency of 1 Hz. Frequency sweep tests were carried out between 0.5 and 100 Hz at 37 °C (human body temperature). Finally, flow curves were performed at 21 °C (operating room temperature) in a shear rate range of 1–100 s^−1^. At least 3 replicates of each sample were made.

### 4.8. Cytotoxicity Tests

Cytotoxicity tests for the microparticles with and without SPIONs/SPIAPTS were performed according to the ISO 10993 standard [59]. The extract method was used with a Vero cell line provided by the American Type Culture Collection (ATCC CCL-81). The extract was produced at a concentration of 5 mg/mL. Each sample was placed in 1 mL of complete culture medium, DMEM (Dulbecco’s modified Eagle’s medium with 1.0 g/L glucose, stable glutamine, and sodium pyruvate, Biowest #L0066) supplemented with penicillin (100 U/mL), streptomycin (100 μg/mL) (Invitrogen, Carlsbad, CA, USA, #15140122), and 10% FBS (fetal bovine serum, S. America origin, Biowest, #S1810) at 37 °C for 48 h. The Vero cells were seeded in 96-well plates at a density of 30 000 cells/cm2. After 24 h, the medium was replaced by the extract and its dilutions (2.5 mg/mL and 1 mg/mL). A negative control corresponded to cells fed with a complete cell culture medium, and a positive control corresponded to cells cultured in a cytotoxic environment caused by the supplementation of the culture medium with 10% dimethyl sulfoxide (DMSO). After 48 h of incubation, the medium was removed from each well and replaced with a resazurin solution containing 50% of the complete medium and 50% of a 0.04 mg/mL resazurin solution in phosphate-buffered saline (PBS). After 3 h of incubation at 37 °C and 5%, the absorbance was measured at 570 and 600 nm. Cell viability is given as a percentage of viable cells in the samples tested relative to the negative control.

### 4.9. Magnetic Hyperthermia Assays

Magnetic hyperthermia measurements were obtained with a NanoScale Biomagnetics D5 Series device (Spain) working at a frequency of 388.5 kHz and a magnetic field intensity of 300 Gauss for 10 min. At least 3 replicates were conducted in each sample, and all samples contained an equal amount of mNPs. Aqueous suspensions of SPIONs (5 mg/mL), aqueous suspensions of SPIAPTS (5 mg/mL), microparticles loaded with mNPs, and magnetic microparticles within Pluronic (17:3) hydrogel in the gel state were analyzed. The specific absorption rate (SAR) indicates the heating efficiency of mNPs through energy absorption with the application of an alternating magnetic field [54] (Equation (4)).

(4) $$SAR\;(W/g)=\frac{C_{NP}\cdot m_{Fe}+C_{l}\cdot m_{l}}{m_{Fe}}{\cdot \left(\frac{dT}{dt}\right)}_{max}$$

In Equation (4), (dT/dt)_max is the maximum derivative of the temperature curve, C_NP is the specific heat of Fe_3_O_4_, Cl is the specific heat of the liquid, mFe is the iron mass, and ml is the mass of the liquid.

### 4.10. Drug Release Studies

To characterize the release profiles of the microparticle/hydrogel composite system with and without mNPs, we used a 1:1 ratio (in mass) of the magnetic microparticles and MB-loaded microparticles. Within a donor–recipient made from a permeable membrane, Pluronic hydrogel composite systems with 2 *w*/*w*% of microparticles were introduced. The recipients of the microparticle/Pluronic were submerged in 10 mL of PBS solutions with pH 6.5 and pH 7.4 at 37 °C. The PBS solutions were kept at 37 °C with orbital agitation. At regular periods (0, 0.25, 1, 3, 6, 24, 48, 72, 144, 192, 240, and 312 h), 2 mL of the PBS was retrieved and replaced with fresh PBS. The 2 mL was then analyzed by UV–VIS spectroscopy using a calibration curve to determine the concentration of MB at each time. Five replicates were made.

The results from the release assays were analyzed using mathematical models. Similarly to our previous work [10], we used a modified Korsmeyer–Peppas (KP) model (Equation (5)) and a modified Peppas–Sahlin (PS) model (Equation (6)) using the DDSolver program [24].

(5) $$Q_{t}=k{\left(t-T_{lag}\right)}^{n}$$

(6) $$Q_{t}=k_{1}{\left(t-T_{lag}\right)}^{m}+k_{2}{\left(t-T_{lag}\right)}^{2m}$$

In Equations (5) and (6), *Q_t_* is the drug concentration released at each time (t), *k* is a structural factor, *n* is related to the release mechanism, and *T_lag_* is the lag time before drug release [58]. The modified Peppas–Sahlin model (PS) uses two constants, *k*_1_ and *k*_2_, of which *k*_1_ correlates with Fickian diffusion and *k*_2_ with case II transport [60].

### 4.11. Statistical Analysis

In different tests, a statistical study was carried out. For this, at least 3 replicates of each sample and assay were performed. A single-factor analysis of variance (ANOVA) was carried out. Then, a series of statistical parameters were calculated, including the mean and standard deviation. In addition, a mean comparison test was performed to detect significant differences (a confidence level of 95%, *p* < 0.05).

## Figures and Tables

**Table 1:** SAR values of samples from Figure 9.

Sample	SAR (W/g)
**SPION suspension**	21.04 ± 0.51
**SPIAPTS suspension**	8.34 ± 0.36
**GG:Alg/SPIONs**	4.14 ± 0.70
**GG:Alg/SPIAPTS**	6.02 ± 1.03

**Table 2:** Parameters obtained from drug-release mathematical fittings of the MB-loaded microparticles within the Pluronic system. The R^2^_adj_ values that compare the experimental and theoretical values are also presented.

		GG:Alg		GG:Alg/SPIONs		GG:Alg/SPIAPTS	
		6.5	7.4	6.5	7.4	6.4	7.4
**KP-T_lag_**	**k**	33.76	36.39	15.78	15.89	8.81	27.78
	**n**	0.21	0.20	0.34	0.33	0.43	0.25
	**T_lag_**	4.98	4.89	3.72	5.31	3.44	5.73
	**R^2^_adj_**	0.97	0.96	0.98	0.99	0.97	0.99
**S-T_lag_**	**k_1_**	14.50	13.71	14.16	11.22	7.51	9.68
	**k_2_**	−0.51	−0.45	−0.46	−0.26	−0.05	−0.24
	**m**	0.53	0.54	0.42	0.45	0.49	0.57
	**T_lag_**	2.91	1.95	2.75	4.12	3.14	3.62
	**R^2^_adj_**	0.99	0.99	0.98	0.99	0.97	0.99

## Data Availability

Data are contained within the article.

## References

[1] Bosio V.E., Cacicedo M.L., Calvignac B., León I., Beuvier T., Boury F., Castro G.R. (2014). Synthesis and characterization of CaCO_3_ –biopolymer hybrid nanoporous microparticles for controlled release of doxorubicin. Colloids Surf. B Biointerfaces.

[2] Sun X., Liu C., Omer A., Yang L.-Y., Ouyang X.-K. (2019). Dual-layered pH-sensitive alginate/chitosan/kappa-carrageenan microbeads for colon-targeted release of 5-fluorouracil. Int. J. Biol. Macromol..

[3] Zhang Y., Jiang C. (2021). Postoperative cancer treatments: In-situ delivery system designed on demand. J. Control. Release.

[4] Fakhari A., Subramony J.A. (2015). Engineered in-situ depot-forming hydrogels for intratumoral drug delivery. J. Control. Release.

[5] Balakrishnan B., Jayakrishnan A., Nair L.S. (2016). Injectable Hydrogels for Biomedical Applications. Injectable Hydrogels for Regenerative Engineering.

[6] Moura M., Gil M., Figueiredo M. (2019). Cisplatin delivery systems based on different drug encapsulation techniques. Eur. Polym. J..

[7] Pontremoli C., Boffito M., Laurano R., Iviglia G., Torre E., Cassinelli C., Morra M., Ciardelli G., Vitale-Brovarone C., Fiorilli S. (2022). Mesoporous Bioactive Glasses Incorporated into an Injectable Thermosensitive Hydrogel for Sustained Co-Release of Sr^2+^ Ions and *N*-Acetylcysteine. Pharmaceutics.

[8] Young S., Wong M., Tabata Y., Mikos A.G. (2005). Gelatin as a delivery vehicle for the controlled release of bioactive molecules. J. Control. Release.

[9] Shymborska Y., Budkowski A., Raczkowska J., Donchak V., Melnyk Y., Vasiichuk V., Stetsyshyn Y. (2023). Switching it Up: The Promise of Stimuli-Responsive Polymer Systems in Biomedical Science. Chem. Rec..

[10] Carrêlo H., Soares P.I.P., Borges J.P., Cidade M.T. (2021). Injectable composite systems based on microparticles in hydrogels for bioactive cargo controlled delivery. Gels.

[11] Yu H., Ning N., Meng X., Chittasupho C., Jiang L., Zhao Y. (2022). Sequential Drug Delivery in Targeted Cancer Therapy. Pharmaceutics.

[12] Naghizadeh Z., Karkhaneh A., Nokhbatolfoghahaei H., Farzad-Mohajeri S., Rezai-Rad M., Dehghan M.M., Aminishakib P., Khojasteh A. (2021). Cartilage regeneration with dual-drug-releasing injectable hydrogel/microparticle system: In vitro and in vivo study. J. Cell. Physiol..

[13] Min Q., Liu J., Yu X., Zhang Y., Wu J., Wan Y. (2019). Sequential delivery of dual growth factors from injectable chitosan-based composite hydrogels. Mar. Drugs.

[14] Desai K.G.H. (2018). Polymeric drug delivery systems for intraoral site-specific chemoprevention of oral cancer. J. Biomed. Mater. Res.—Part B Appl. Biomater..

[15] Wang A.Z., Langer R., Farokhzad O.C. (2012). Nanoparticle Delivery of Cancer Drugs. Annu. Rev. Med..

[16] Schneider M.G.M., Martín M.J., Otarola J., Vakarelska E., Simeonov V., Lassalle V., Nedyalkova M. (2022). Biomedical Applications of Iron Oxide Nanoparticles: Current Insights Progress and Perspectives. Pharmaceutics.

[17] Musielak M., Piotrowski I., Suchorska W.M. (2019). Superparamagnetic iron oxide nanoparticles (SPIONs) as a multifunctional tool in various cancer therapies. Rep. Pract. Oncol. Radiother..

[18] Neuwelt A., Sidhu N., Hu C.A.A., Mlady G., Eberhardt S.C., Sillerud L.O. (2015). Iron-based superparamagnetic nanoparticle contrast agents for MRI of infection and inflammation. Am. J. Roentgenol..

[19] Finotelli P.V., Da Silva D., Sola-Penna M., Rossi A.M., Farina M., Andrade L.R., Takeuchi A.Y., Rocha-Leão M.H. (2010). Microcapsules of alginate/chitosan containing magnetic nanoparticles for controlled release of insulin. Colloids Surf. B Biointerfaces.

[20] Matos R.J.R., Chaparro C.I.P., Silva J.C., Valente M.A., Borges J.P., Soares P.I.P. (2018). Electrospun composite cellulose acetate/iron oxide nanoparticles non-woven membranes for magnetic hyperthermia applications. Carbohydr. Polym..

[21] Alpdemir Ş., Vural T., Kara G., Bayram C., Haberal E., Denkbaş E.B. (2020). Magnetically responsive, sorafenib loaded alginate microspheres for hepatocellular carcinoma treatment. IET Nanobiotechnol..

[22] Xue W., Liu X.-L., Ma H., Xie W., Huang S., Wen H., Jing G., Zhao L., Liang X.-J., Fan H.M. (2018). AMF responsive DOX-loaded magnetic microspheres: Transmembrane drug release mechanism and multimodality postsurgical treatment of breast cancer. J. Mater. Chem. B.

[23] Carrêlo H., Escoval A.R., Soares P.I.P., Borges J.P., Cidade M.T. (2022). Injectable Composite Systems of Gellan Gum:Alginate Microparticles in Pluronic Hydrogels for Bioactive Cargo Controlled Delivery: Optimization of Hydrogel Composition based on Rheological Behavior. Fluids.

[24] Vu-Quang H., Vinding M.S., Nielsen T., Ullisch M.G., Nielsen N.C., Nguyen D.-T., Kjems J. (2019). Pluronic F127-folate coated super paramagenic iron oxide nanoparticles as contrast agent for cancer diagnosis in magnetic resonance imaging. Polymers.

[25] Rodríguez-López J., Shum H.C., Elvira L., de Espinosa F.M., Weitz D.A. (2013). Fabrication and manipulation of polymeric magnetic particles with magnetorheological fluid. J. Magn. Magn. Mater..

[26] Li N., Zhao L., Qi L., Li Z., Luan Y. (2016). Polymer assembly: Promising carriers as co-delivery systems for cancer therapy. Prog. Polym. Sci..

[27] Carrêlo H., Cidade M.T., Borges J.P., Soares P. (2023). Gellan gum/alginate microparticles as drug delivery vehicles: DOE production optimization and drug delivery. Pharmaceuticals.

[28] Halim N.F.A., Majid S.R., Arof A.K., Kajzar F., Pawlicka A. (2012). Gellan Gum-LiI gel polymer electrolytes. Mol. Cryst. Liq. Cryst..

[29] Khampieng T., Aramwit P., Supaphol P. (2015). Silk sericin loaded alginate nanoparticles: Preparation and anti-inflammatory efficacy. Int. J. Biol. Macromol..

[30] Noor I., Majid S., Arof A., Djurado D., Neto S.C., Pawlicka A. (2012). Characteristics of gellan gum–LiCF_3_SO_3_ polymer electrolytes. Solid State Ionics.

[31] Patel N., Lalwani D., Gollmer S., Injeti E., Sari Y., Nesamony J. (2016). Development and evaluation of a calcium alginate based oral ceftriaxone sodium formulation. Prog. Biomater..

[32] Soares P.I.P., Lochte F., Echeverria C., Pereira L.C.J., Coutinho J.T., Ferreira I.M.M., Novo C.M.M., Borges J.P.M.R. (2015). Thermal and magnetic properties of iron oxide colloids: Influence of surfactants. Nanotechnology.

[33] Kloster G.A., Muraca D., Londoño O.M., Pirota K.R., Mosiewicki M.A., Marcovich N.E. (2020). Alginate based nanocomposites with magnetic properties. Compos. Part A: Appl. Sci. Manuf..

[34] Jana S., Das A., Nayak A.K., Sen K.K., Basu S.K. (2013). Aceclofenac-loaded unsaturated esterified alginate/gellan gum microspheres: In vitro and in vivo assessment. Int. J. Biol. Macromol..

[35] Villa S., Riani P., Locardi F., Canepa F. (2016). Functionalization of Fe_3_O_4_ NPs by silanization: Use of amine (APTES) and thiol (MPTMS) silanes and their physical characterization. Materials.

[36] Stanković A., Mihailović J., Mirković M., Radović M., Milanović Z., Ognjanović M., Janković D., Antić B., Mijović M., Vranješ-Đurić S. (2020). Aminosilanized flower-structured superparamagnetic iron oxide nanoparticles coupled to 131I-labeled CC49 antibody for combined radionuclide and hyperthermia therapy of cancer. Int. J. Pharm..

[37] Shukla S., Jadaun A., Arora V., Sinha R.K., Biyani N., Jain V. (2015). In vitro toxicity assessment of chitosan oligosaccharide coated iron oxide nanoparticles. Toxicol. Rep..

[38] Khalkhali M., Rostamizadeh K., Sadighian S., Khoeini F., Naghibi M., Hamidi M. (2015). The impact of polymer coatings on magnetite nanoparticles performance as MRI contrast agents: A comparative study. DARU J. Pharm. Sci..

[39] Mun S., Kim H.C., Yadave M., Kim J. (2015). Graphene oxide–gellan gum–sodium alginate nanocomposites: Synthesis, characterization, and mechanical behavior. Compos. Interfaces.

[40] (2009). Biological Evaluation of Medical Devices Part 5: Tests for In Vitro Cytotoxicity.

[41] Amjadi S., Hamishehkar H., Ghorbani M. (2019). A novel smart PEGylated gelatin nanoparticle for co-delivery of doxorubicin and betanin: A strategy for enhancing the therapeutic efficacy of chemotherapy. Mater. Sci. Eng. C Mater. Biol. Appl..

[42] Nie S., Hsiao W.L.W. (2011). Thermoreversible Pluronic ® F127-based hydrogel containing liposomes for the controlled delivery of paclitaxel: In vitro drug release, cell cytotoxicity, and uptake studies. Int. J. Nanomed..

[43] Diniz I.M.A., Chen C., Xu X., Ansari S., Zadeh H.H., Marques M.M., Shi S., Moshaverinia A. (2015). Pluronic F-127 hydrogel as a promising scaffold for encapsulation of dental-derived mesenchymal stem cells. J. Mater. Sci. Mater. Med..

[44] de Melo Santana B., Pieretti J.C., Gomes R.N., Cerchiaro G., Seabra A.B. (2022). Cytotoxicity towards Breast Cancer Cells of Pluronic F-127/Hyaluronic Acid Hydrogel Containing Nitric Oxide Donor and Silica Nanoparticles Loaded with Cisplatin. Pharmaceutics.

[45] García-Couce J., Tomás M., Fuentes G., Que I., Almirall A., Cruz L.J. (2022). Chitosan/Pluronic F127 Thermosensitive Hydrogel as an Injectable Dexamethasone Delivery Carrier. Gels.

[46] Pragatheeswaran A.M., Chen S.B., Chen C.-F., Chen B.-H. (2014). Micellization and gelation of PEO-PPO-PEO binary mixture with non-identical PPO block lengths in aqueous solution. Polymer.

[47] Sánchez-Cid P., Jiménez-Rosado M., Rubio-Valle J.F., Romero A., Ostos F.J., Benhnia M.R.-E., Perez-Puyana V. (2022). Biocompatible and Thermoresistant Hydrogels Based on Collagen and Chitosan. Polymers.

[48] Gonçalves L.C., Seabra A.B., Pelegrino M.T., de Araujo D.R., Bernardes J.S., Haddad P.S. (2017). Superparamagnetic iron oxide nanoparticles dispersed in Pluronic F127 hydrogel: Potential uses in topical applications. RSC Adv..

[49] Jain T.K., Morales M.A., Sahoo S.K., Leslie-Pelecky D.L., Labhasetwar V. (2005). Iron oxide nanoparticles for sustained delivery of anticancer agents. Mol. Pharm..

[50] Chandra S., Mehta S., Nigam S., Bahadur D. (2010). Dendritic magnetite nanocarriers for drug delivery applications. New J. Chem..

[51] Ota S., Takemura Y. (2019). Characterization of Néel and Brownian Relaxations Isolated from Complex Dynamics Influenced by Dipole Interactions in Magnetic Nanoparticles. J. Phys. Chem. C.

[52] Liu X., Wang Y., Zhu W., Li G., Ma X., Zhang Y., Chen S., Tiwari S., Shi K., Zhang S. (2020). Comprehensive understanding of magnetic hyperthermia for improving antitumor therapeutic efficacy. Theranostics.

[53] Gonçalves A., Almeida F.V., Borges J.P., Soares P.I.P. (2021). Incorporation of dual-stimuli responsive microgels in nanofibrous membranes for cancer treatment by magnetic hyperthermia. Gels.

[54] Monks P., Wychowaniec J.K., McKiernan E., Clerkin S., Crean J., Rodriguez B.J., Reynaud E.G., Heise A., Brougham D.F. (2021). Spatiotemporally Resolved Heat Dissipation in 3D Patterned Magnetically Responsive Hydrogels. Small.

[55] Heo J.Y., Noh J.H., Park S.H., Ji Y.B., Ju H.J., Kim D.Y., Lee B., Kim M.S. (2019). An injectable click-crosslinked hydrogel that prolongs dexamethasone release from dexamethasone-loaded microspheres. Pharmaceutics.

[56] Soares P.I., Laia C.A., Carvalho A., Pereira L.C., Coutinho J.T., Ferreira I.M., Novo C.M., Borges J.P. (2016). Iron oxide nanoparticles stabilized with a bilayer of oleic acid for magnetic hyperthermia and MRI applications. Appl. Surf. Sci..

[57] Liu Y., Li Y., Li X.-M., He T. (2013). Kinetics of (3-aminopropyl)triethoxylsilane (APTES) silanization of superparamagnetic iron oxide nanoparticles. Langmuir.

[58] Zhang Y., Huo M., Zhou J., Zou A., Li W., Yao C., Xie S. (2010). DDSolver: An add-in program for modeling and comparison of drug dissolution profiles. AAPS J..

[59] (2018). Biological Evaluation of Medical Devices Part 1: Evaluation and Testing within a Risk Management Process.

[60] Arifin D.Y., Lee L.Y., Wang C.-H. (2006). Mathematical modeling and simulation of drug release from microspheres: Implications to drug delivery systems. Adv. Drug Deliv. Rev..

